# Supplementary material for: Molecular Cues for Phenological Events in the Flowering Cycle in Avocado
Source: Plants (Basel). 2023 Jun 13;12(12):2304. doi: 10.3390/plants12122304 (PMC10305662; doi:10.3390/plants12122304)
Supplement: Supplementary file 1 [file plants-12-02304-s001.zip › plants-2428931-supplementary.pdf]

Supplementary Tables

Table S1: *FT*-related transcripts details used for alignment and phylogenetic tree.

No.	Name	*Organism*	Seq detail
1	AtFT	*Arabiidopsis thaliana*	AT1G65480
2	MsFT	*Medicago sativa*	AEO16612.1
3	ZjFT	*Ziziphus jujuba*	ANG60748.1
4	VvFT	*Vitis vinifera*	ABI99465.1
5	BnFT	*Brassica napus*	ACY03405.1
6	RcFT	*Rosa chinensis*	CBY25182.1
7	PmFT	*Prunus mume*	CBY25181.1
8	GmFT	*Glycine max*	ABZ80360.1
9	TaeFT	*Triticum aestivum*	ACA25439.1
10	JcFT	*Jatropha curcas*	AID51385.1
11	MiFT	*Mangifera indica*	AGA19021.1
12	StFT	*Solanum tuberosum*	NP_001274897.1
13	MdFT	*Malus domestica*	ABF84010.1
14	PaFT	*Persea americana*	AIG92770.1
15	PnFT	*Populus nigra*	BAG12904.1
16	AaFT	*Arabis alpina*	KFK30313.1
17	CsiFT	*Camellia sinensis*	BAM83573.1
18	CuFT	*Citrus unshiu*	BAF96645.1
19	BnTFL1	*Brassica napus*	ATQ37956.1
20	MdTFL1	*Malus domestica*	ACD69429.1
21	AhTFL1	*Arachis hypogaea*	AFP33421.1
22	MtTFL1	*Medicago truncatula*	KEH17361.1
23	ClTFL1	*Citrus limon*	AWW25018.1
24	AtTFL1	*Arabidopsis thaliana*	ANS12868.1
25	PpeTFL1	*Prunus persica*	ADL62867.1
26	MiTFL1	*Mangifera indica*	AGA19026.1

27	PnTFL1	*Populus nigra*	BAG12897.1
28	AtCEN	*Arabidopsis thaliana*	AT2G27550
29	TcCEN	*Theobroma cacao*	EOY33468.1
30	AmCEN	*Antirrhinum majus*	CAC21564.1
31	HvCEN	*Hordeum vulgare*	AFV67450.1
32	MdCEN2	*Malus domestica*	P_001280813.1
33	MdCEN1	*Malus domestica*	BAG31958.1
34	AtMFT	*Arabidopsis thaliana*	AT1G18100
35	BnMFT	*Brassica napus*	XP_013723319.1
36	RcMFT	*Rosa chinensis*	XP_024169398.1
37	MtMFT	*Medicago truncatula*	XP_003631075.2
38	CsMFT	*Citrus sinensis*	XP_006467912.1
39	ZjMFT	*Ziziphus jujuba*	XP_015886852.1
40	ClMFT	*Citrus limon*	AWW25016.1
41	AhMFT	*Arachis hypogaea*	XP_025639284.1
42	SlMFT	*Solanum lycopersicum*	XP_004235817.1
43	JcMFT.2	*Jatropha curcas*	AID51395.1
44	JcMFT.1	*Jatropha curcas*	AHG97810.1
45	TaeMFT	*Triticum aestivum*	BAK78909.1
46	AtBFT	*Arabidopsis thaliana*	AT5G62040.1
47	ClBFT	*Citrus limon*	AWW25017.1
48	BnBFT	*Brassica napus*	XP_013723459.1
49	MdMFTb	*Malus domestica*	AGX15187.1

Supplementary Table S2: MADS-box (AP1, SEP, SOC1, SVP/DAM) related transcripts details used for alignment and phylogenetic tree.

No.	Name	Species	Sequence details
1	AtAP1	*Arabidopsis thaliana*	AT1G69120.1
2	MiAP1	*Mangifera indica*	ACS45103.1
3	PtAP1.1	*Populus trichocarpa*	AAT39554.1
4	StAP1	*Solanum tuberosum*	ADA77531.1
5	MdAP1	*Malus domestica*	ACD69426.1
6	VuAP1	*Vigna unguiculata*	BAJ22385.1
7	NnAP1	*Nelumbo nucifera*	AGY54940.1
8	JcAP1	*Jatropha curcas*	AKM16736.1
9	VvAP1	*Vitis vinifera*	NP_001268210.1
10	CsAP1	*Citrus sinensis*	NP_001275828.1
11	PaAP1	*Persea americana*	ABD62862.1
12	AtFUL	*Arabidopsis thaliana*	AT5G60910.1
13	VvFUL	*Vitis vinifera*	ACZ26529.1
14	AtSOC1	*Arabidopsis thaliana*	AT2G45660.1
15	PtSOC1	*Populus trichocarpa*	XP_006383341.2
16	NtSOC1-like	*Nicotiana tabacum*	NP_001312958.1
17	MdSOC1-like	*Malus domestica*	NP_001280844.1
18	PmSOC1-like	*Prunus mume*	NP_001306730.1
19	GmSOC1	*Glycine max*	ABC75835.1
20	AaSOC1	*Arabis alpina*	AEH43355.1
21	CsiSOC1	*Camellia sinensis*	ALS54681.1
22	MiSCO1	*Mangifera indica*	ADX97324.1
23	ParSOC1	*Prunus armeniaca*	AGD88524.1
24	EgrSOC1	*Eucalyptus grandis*	XP_018716234.1
25	AtSVP	*Arabidopsis thaliana*	AT2G22540.1

26	BnSVP	*Brassica napus*	AFM77910.1
27	JcSVP	*Jatropha curcas*	XP_012081656.1
28	CpSVP	*Carica papaya*	XP_021897605.1
29	CsSVP	*Citrus sinensis*	XP_006472471.1
30	PmSVP	*Prunus mume*	NP_001313437.1
31	AaSVP	*Arabis alpina*	KFK32803.1
32	GmSVP	*Glycine max*	NP_001240951.1
33	PpDAM1	*Prunus persica*	ABJ96361.2
34	MdDAM2	*Malus domestica*	AOA32866.1
35	MdDAM1	*Malus domestica*	AOA32865.1
36	PmDAM1	*Prunus mume*	BAK78921.1
37	PmDAM2	*Prunus mume*	BAK78922.1
38	AtSEP2	*Arabidopsis thaliana*	AT3G02310.1
39	AtSEP1	*Arabidopsis thaliana*	AT5G15800.2
40	AtSEP3	*Arabidopsis thaliana*	AT1G24260.2
41	AtSEP4	*Arabidopsis thaliana*	AT2G03710.1
42	BnSEP1	*Brassica napus*	XP_013720978.1
43	PtSEP2	*Populus trichocarpa*	XP_024444692.1
44	CsSEP1	*Citrus sinensis*	XP_006482430.1
45	ZjSEP1	*Ziziphus jujuba*	XP_024928213.1
46	ZjSEP3	*Ziziphus jujuba*	XP_024928665.1
47	AaSEP3	*Arabis alpina*	KFK44483.1
48	MdSEP1	*Malus domestica*	NP_001280893.1
49	EgrSEP1	*Eucalyptus grandis*	NP_001289642.1

Table S3: CO-related transcripts details used for alignment and phylogenetic tree.

	Name	*Species*	Seq. detail
1	StCO	*Solanum tuberosum*	NP_001274795.1
2	AtCO	*Arabidopsis thaliana*	AT5G15840.1
3	SlCO2	*Solanum lycopersicum*	AAS67378.1
4	SlCO1	*Solanum lycopersicum*	AAS67377.1
5	NtCO	*Nicotiana tabacum*	AEJ84000.1
6	GmCO	*Glycine max*	ACJ65311.1
7	MiCO	*Mangifera indica*	ADX97322.1
8	PdCO2	*Populus deltoides*	AAS00055.1
9	MdCOL4	*Malus domestica*	NP_001280817.1
10	MdCOL1	*Malus domestica*	AAC99309.1
11	MdCOL2	*Malus domestica*	AAC99310.1
12	MtCO1	*Medicago truncatula*	XP_013447078.1
13	AtCOL5	*Arabidopsis thaliana*	AT5G57660.1
14	AtCOL4	*Arabidopsis thaliana*	AT5G24930.1
15	AtCOL1	*Arabidopsis thaliana*	AT5G15850.1
16	AtCOL2	*Arabidopsis thaliana*	AT3G02380.1
17	AtCOL3	*Arabidopsis thaliana*	AT2G24790.1

Supplementary Table S4: New gene transcript identified from in-house transcriptome/genome data. Full length Coding region of these transcripts were predicted using Geneious software and were then submitted to NCBI repository. The integrity of each transcript's protein domain was validated using phmmr online prediction tool.

**Gene**	**NCBI submission**	**phmmr (pfam.xfam.org) protein domain validation results**
PaAGL4	Submitted, pending approval	SRF, K BOX
PaMFTa	Submitted, pending approval	Phosphatidylethanolamine-binding protein
PaMFTb	Submitted, pending approval	Phosphatidylethanolamine-binding protein
PaSOC1b	Submitted, pending approval	SRF, K BOX
PaFUL	Submitted, pending approval	SRF, K BOX
PaCOa	Submitted, pending approval	B-box zinc finger, CCT motif
PaCOb	Submitted, pending approval	B-box zinc finger, CCT motif
PaDAM	Submitted, pending approval	SRF, K BOX
PaCYCD3;1	Submitted, pending approval	Cyclin, N-terminal domain and C-terminal domain
MciSEP3	Submitted, pending approval	SRF, K BOX
MciMFT	Submitted, pending approval	Phosphatidylethanolamine-binding protein
MciCOa	Submitted, pending approval	B-box zinc finger, CCT motif

MciCOb	Submitted, pending approval	B-box zinc finger, CCT motif
MciFT	Submitted, pending approval	Phosphatidylethanolamine-binding protein
MciCEN	Submitted, pending approval	Phosphatidylethanolamine-binding protein
MciSOC1b	Submitted, pending approval	SRF, K BOX
MciTFL	Submitted, pending approval	Phosphatidylethanolamine-binding protein
MciDAM	Submitted, pending approval	SRF, K BOX
MciCYCD2;1	Submitted, pending approval	Cyclin, N-terminal domain and C-terminal domain
MciFUL	Submitted, pending approval	SRF, K BOX

Supplementary Table S5: Primers used to amplify transcripts.

No.	Gene	F Primer	R Primer
1	*PaAGL4*	AACTACAGTCGCCAAGCTCC	TCCATCCAGGAACGAAACCG
2	*PaMFTa*	ATCAAGCCCTCCATTGCCCA	CGCATCCGGGTCTGTCATCA
3	*PaMFTb*	AGCCGGTTCCTTCTGTGCAT	TTTCAACACCCGCACGTTCG
4	*PaSOC1b*	AAGCAGGCAAGTGACGTTCT	TTTCCATCCCTTGATTGGAG
5	*PaFUL*	CCTTCTCAAGAAAGCGAACG	TATTCGGTGAGCTTGCCTCT
6	*PaCOa*	TTCAGATGCCACACCAGTTC	GCATAAGCCTTCCTTGAAGC
7	*PaCOb*	ACGCTTTGCCAAGAGAACAG	TACAATCCCGTATCCGCTTT
8	*PaDAM*	CAGGTAGCAGAAAAGGGCCA	CTAGCACGCGACTTAGTCCC
9	*PaCYCD3;1*	CAGGTAGCAGAAAAGGGCCA	CTAGCACGCGACTTAGTCCC
10	*PaPP2AA3*	AATTGGGGCCCAGATGAAGG	CTCTGGTGCACTTGGGTAGG
11	*PaGAPDH*	TGGGAAACTTACAGGAATGG	GTCACCCACAAAGTCAGTAGAA
12	*PaEF1a*	ATCAAGCGTGGGTTTGTTGC	TACCCGTTGCCAATCTGACC
